# Employing 454 amplicon pyrosequencing to reveal intragenomic divergence in the internal transcribed spacer rDNA region in fungi

**DOI:** 10.1002/ece3.586

**Published:** 2013-05-08

**Authors:** Daniel L Lindner, Tor Carlsen, R Henrik Nilsson, Marie Davey, Trond Schumacher, Håvard Kauserud

**Affiliations:** 1 US Forest Service, Northern Research Station, Center for Forest Mycology ResearchOne Gifford Pinchot Drive, Madison, Wisconsin; 2 Microbial Evolution Research Group (MERG), Department of Biology, University of OsloPO Box 1066 Blindern, NO-0316, Oslo, Norway; 3 Department of Biological and Environmental Sciences, University of GothenburgBox 461, 405 30, Gothenburg, Sweden; 4 Department of Ecology and Natural Resource Management, Norwegian University of Life SciencesPO Box 5003, NO-1432, Ås, Norway

**Keywords:** Environmental sequencing, operational taxonomic units, pyrosequencing, species richness

## Abstract

The rDNA internal transcribed spacer (ITS) region has been accepted as a DNA barcoding marker for fungi and is widely used in phylogenetic studies; however, intragenomic ITS variability has been observed in a broad range of taxa, including prokaryotes, plants, animals, and fungi, and this variability has the potential to inflate species richness estimates in molecular investigations of environmental samples. In this study 454 amplicon pyrosequencing of the ITS1 region was applied to 99 phylogenetically diverse axenic single-spore cultures of fungi (Dikarya: Ascomycota and Basidiomycota) to investigate levels of intragenomic variation. Three species (one Basidiomycota and two Ascomycota), in addition to a positive control species known to contain ITS paralogs, displayed levels of molecular variation indicative of intragenomic variation; taxon inflation due to presumed intragenomic variation was ≈9%. Intragenomic variability in the ITS region appears to be widespread but relatively rare in fungi (≈3–5% of species investigated in this study), suggesting this problem may have minor impacts on species richness estimates relative to PCR and/or pyrosequencing errors. Our results indicate that 454 amplicon pyrosequencing represents a powerful tool for investigating levels of ITS intragenomic variability across taxa, which may be valuable for better understanding the fundamental mechanisms underlying concerted evolution of repetitive DNA regions.

## Introduction

The internal transcribed spacer (ITS) region of nuclear ribosomal DNA is the most commonly sequenced region in fungi and is used in fungal systematics to define species, to infer phylogenetic relationships, and for identification (DNA barcoding) of fruiting bodies, cultures, and DNA in environmental samples (Horton and Bruns 2001; Peay et al. 2008; Begerow et al. 2010). The ITS region has recently been proposed as the universal barcode for all fungi (Schoch et al. 2012). Although mycologists rely heavily on ITS to define and detect species and to understand fungal evolution, there are many long-recognized problems with using this region. Problems range from a lack of interspecific variation in some groups of fungi, especially some Ascomycota (Rehner and Buckley 2005; Balajee et al. 2007; Rojas et al. 2010), to an abundance of variation among individuals within populations (Kårén et al. 1997; Kauserud and Schumacher 2002; Nilsson et al. 2008; Blaalid et al. 2013). These problems are not unique to the ITS region and it is unlikely that any single, short DNA region includes levels of molecular variation suitable for separating species across a phylogenetic group as broad as kingdom Fungi, with an estimated 1.5–5.1 million extant species (Hawksworth 2001; Schmit and Mueller 2007; Blackwell 2011).

However, one problem that is relatively unique to rDNA regions, including the ITS region, is the possibility for significant intragenomic (within-individual) variability. This potential arises because the ribosomal tandem array occurs at high copy number, which in fungi can range from approximately 45 to 200 copies per genome and span several chromosomes (Maleszka and Clark-Walker 1990; Ganley and Kobayashi 2007). Intragenomic ITS variability has been observed in a wide range of taxa, including prokaryotes, plants, animals, and fungi (Feliner et al. 2004; Wörheide et al. 2004; Stewart and Cavanaugh 2007; Simon and Weiss 2008; James et al. 2009; Vydryakova et al. 2012). In one recent case, intragenomic ITS variation was noted in the fungal genus *Laetiporus*, a group of brown-rot polypores in the Antrodia clade (Lindner and Banik 2011). Intragenomic variation in this group was found to inflate estimates of species richness and to complicate phylogenetic investigations when cloned ITS sequences rather than ITS sequences obtained by direct Sanger sequencing were analyzed. Unfortunately it is not known how widespread this phenomenon is in kingdom Fungi. If such intragenomic variation is common it will cause significant problems with the analysis of environmental sequencing data. These problems could be especially severe with high-throughput next-generation sequencing methods (e.g., 454 pyrosequencing, Illumina, and IonTorrent), where even low-frequency ITS paralogs will be detected.

Our aim was to explore levels of intragenomic divergence in the Dikarya (Ascomycota and Basidiomycota) using large-scale sequencing of ITS1 amplicons derived from axenic single-spore cultures. *Laetiporus cincinnatus*, a species known to contain significant intragenomic variation (Lindner and Banik 2011), was included as a positive control. A wide range of phylogenetically diverse Basidiomycota and Ascomycota single-spore cultures were chosen from culture collections and the ITS1 region was amplified and subjected to 454 pyrosequencing (Margulies et al. 2005).

## Materials and Methods

### Fungal cultures

One hundred and twenty-seven single-spore cultures from diverse phylogenetic lineages in the Dikarya (Ascomycota and Basidiomycota) were originally screened for use in this study. Of these, 99 produced >100 pyrosequencing reads following initial data filtering (see methods below) and were included in the final dataset; 44 were Ascomycota and 55 were Basidiomycota (Appendix). Cultures were obtained from the culture collections of the Center for Forest Mycology Research (CFMR), maintained by the US Forest Service, Northern Research Station in Madison, WI; the ARON culture collection at the Department of Biology, University of Oslo; the Norwegian Veterinary Institute; and from the Norwegian Forest and Landscape Institute culture collections. All Basidiomycota were checked for, and found to lack, clamp connections, one potential sign of a dikaryotic mycelium.

### Molecular methods

DNA was extracted from the axenic cultures following a 2% CTAB (hexadecyl-trimethyl-ammonium bromide) miniprep method described by Murray and Thompson (1980) with minor modifications: DNA was resuspended in 60-μL distilled sterile H_2_O at the final step of extraction. Samples were prepared for 454 pyrosequencing by performing nested PCR amplification using the fungal-specific primers ITS1F and ITS4 (White et al. 1990; Gardes and Bruns 1993) in the first step, and fusion primers including ITS5 and ITS2 (White et al. 1990) in the nested step. Fusion primers were constructed by adding 16 different unique 10 bp tags (Technical bulletin 005-2009, Roche Diagnostics Corp., Basel, Switzerland) and 454 pyrosequencing Titanium adaptors A and B to ITS5 and ITS2, respectively. The same tags were added to both forward and reverse primers. All PCR reactions were performed in three parallels for all samples for both PCR steps. PCR was performed on an MJ thermal cycler PTC-200 in 20-μL reactions containing 2-μL template DNA and 18-μL reaction mix. Final concentrations were 0.10 mmol/L dNTP mix, 0.125 μmol/L of each primer, and 0.5 units polymerase (Phusion Hot Start II, Finnzymes, Vantaa). The PCR amplification program was as follows: 30 sec at 98°C, followed by 20 cycles of 10 sec at 98°C, 20 sec at 50°C, 20 sec at 72°C, and a final extension step at 72°C for 7 min before storage at −20°C. The nested PCR was run with the same reaction concentrations and amplification program, but with a 50× diluted PCR mix as a template. After normalization of DNA concentration using the SequalPrep™ Normalization Plate (96) Kit following the manufacturer's protocol (Invitrogen, CA), PCR products were pooled into 8 equimolar amplicon libraries and cleaned with Wizard® SV Gel and PCR Clean-Up System (Promega, Madison, WI). The 454 Titanium sequencing of the tagged amplicons was performed at the Norwegian High-Throughput Sequencing Centre (http://www.sequencing.uio.no) using a 454 plate divided into eight compartments.

### Bioinformatics analyses

As an initial filter, we removed all sequences with more than two errors in the primer sequence; with one or more errors in the tag sequence; with one or more DNA ambiguity symbols (N); or with an overall length of less than 150 bases. Reads with noncompatible tag combinations (Carlsen et al. 2012) also were removed. Sequence data were not denoized (e.g., Quince et al. 2011) so as to retain PCR and sequencing errors in addition to intragenomic ITS variation. Based on tag information, the sequences were split into 127 datasets representing the various single-spore cultures plus two negative controls. Twenty-eight datasets were discarded from further analyses due to a low number of reads (<100), leaving 99 species in the final dataset. Alignments were constructed in MAFFT 6.903 (Katoh and Toh 2008) for all datasets using the default (auto) strategy, which typically resulted in the FFT-NS-1 or FFT-NS-2 algorithm being selected. Manual inspection and BLAST searches (Altschul et al. 1997) of GenBank (Benson et al. 2012) also identified “contaminant sequences” in some datasets that represented species from the other datasets. These were interpreted as sequences that had switched tags at both ends (see Carlsen et al. 2012) and were excluded from the analysis. The final MAFFT alignments of the 99 accepted datasets were analyzed in DnaSP (Librado and Rozas 2009), where descriptive molecular variation statistics were calculated, including number of reads, number of alignment sites, number of haplotypes, haplotype diversity, nucleotide diversity (pi), and average number of nucleotide differences (k).

Using single linkage clustering as implemented in BLASTCLUST (cf. Altschul et al. 1997) all datasets were clustered using 85% sequence coverage and either 97% or 99% sequence similarity. The total number of clusters as well as nonsingleton clusters was calculated. In eight species (*Armillaria* cf. *novae-zelandiae* HHB15567, *Aspergillus* sp. VI05307, *Annulohypoxylon multiforme* 1967-10_ss-1, *L. cincinnatus* HHB15746, *Laetiporus conifericola* AK1, *Laetiporus huroniensis* HMC1, *Laetiporus sulphureus* DA41, and Polyporales sp. HHB9461, hereafter referred to without collection numbers), data were explored further using neighbor-joining analyses as implemented in MEGA (Tamura et al. 2007) with the Jukes–Cantor model of evolution and uniform rate variation among sites implemented.

## Results

**Figure 1:**
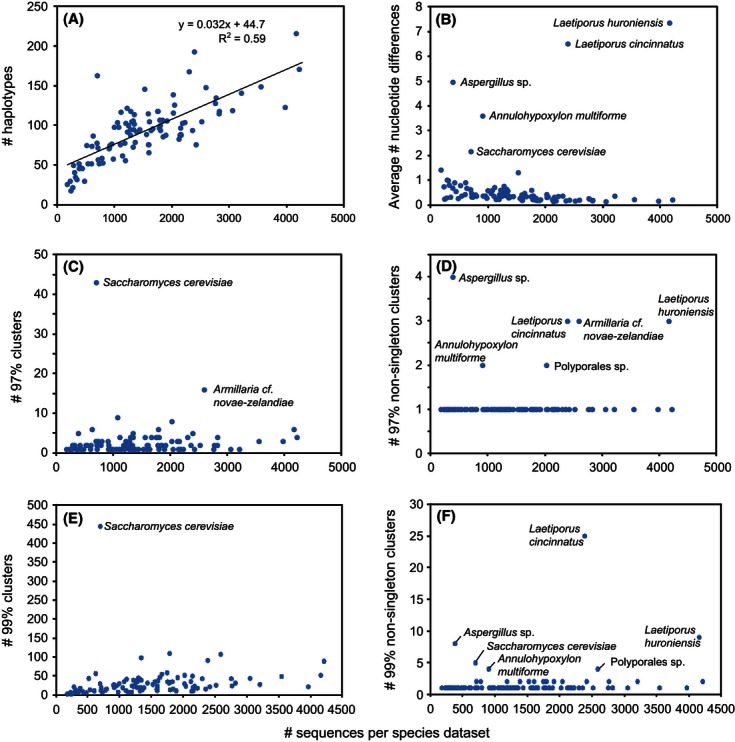
Molecular variation in the internal transcribed spacer (ITS1) amplified from 99 fungal species and plotted against sequencing depth. (A) Number of ITS1 haplotypes in each dataset. (B) Average number of nucleotide differences between ITS1 sequences in each dataset. (C) Number of sequence clusters obtained from each dataset using BLASTCLUST and a 97% sequence similarity cutoff. (D) Number of nonsingleton sequence clusters obtained from each dataset using BLASTCLUST and a 97% sequence similarity cutoff. (E) Number of sequence clusters obtained from each dataset using BLASTCLUST and a 99% sequence similarity cutoff. (F) Number of nonsingleton sequence clusters obtained from each dataset using BLASTCLUST and a 99% sequence similarity cutoff.

After filtering, a total of 148,046 sequences were analyzed from the 99 isolates, yielding 9086 individual haplotypes (i.e., the number of clusters at 100% sequence similarity; Appendix). The number of reads per species ranged from 176 to 4212 with an average of 1495 reads per species. A strong positive correlation was observed between number of haplotypes per species and sequencing depth (Fig. 1A). However, the average number of nucleotide differences per species did not correlate with sequencing depth (Fig. 1B). There was a weak positive correlation between the total number of clusters detected per species and sequencing depth both at the 97% (Fig. 1C) and 99% (Fig. 1E) clustering level. However, when the comparison was restricted to nonsingleton clusters, no relationship was detected between the number of clusters and sequencing depth at 97% (Fig. 1D) or 99% (Fig. 1F). In the full dataset of 99 taxa, 97% clustering of sequences produced 286 clusters. When excluding the singletons, 110 clusters were retained (Appendix). Even at a 99% sequence clustering level, 79% of the species included only one cluster when excluding singletons. In 92 of the 99 species, between 99.6% and 100% of the sequences were assigned to a single cluster by the clustering process (Appendix). Only in three species (*Aspergillus* sp., *L. cincinnatus*, and *L. huroniensis*) were more than 1.1% of the sequences affiliated with cluster(s) other than the most frequent.

**Figure 2:**
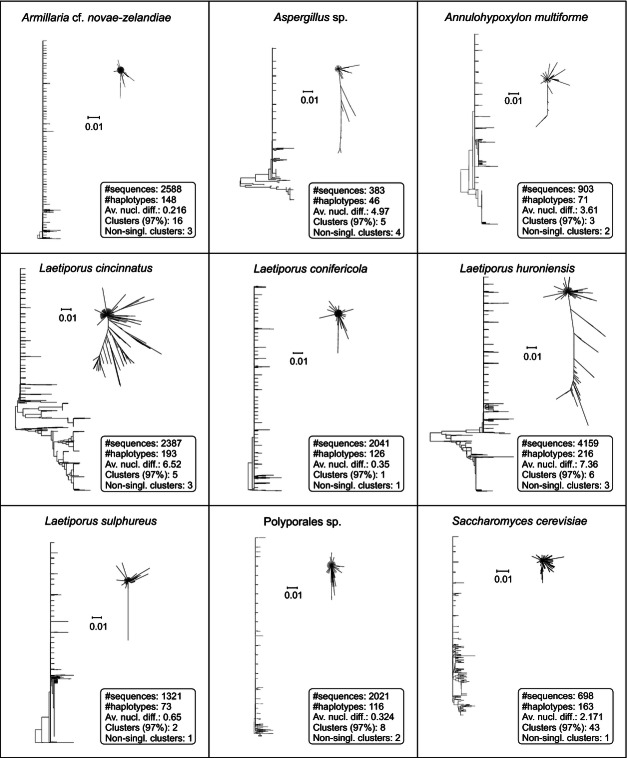
Neighbor-joining trees illustrating sequence variation in the ITS1 alignments in species with varying levels of molecular variation. *Armillaria* cf. *novae-zelandiae*, *Aspergillus* sp., *Annulohypoxylon multiforme*, *Laetiporus cincinnatus*, *L. huroniensis*, Polyporales sp., and *Saccharomyces cerevisiae* are included as species with high levels of variation, whereas *Laetiporus conifericola* and *L. sulphurensis* are included as typical examples representing species with lower levels of variation. Both midpoint rooted and unrooted trees are shown for all taxa. Similar scales are used across all trees to enable direct comparisons. We hypothesize that the star-shaped unrooted trees observed for *Armillaria* cf*. novae-zelandiae, L. conifericola*, *L. sulphurensis,* Polyporales sp., and *Saccharomyces cerevisiae* are due to PCR and sequencing errors, whereas the more complex trees for *Aspergillus* sp., *Annulohypoxylon multiforme, L. cincinnatus*, and *L. huroniensis* are due to intragenomic variation.

As expected, the *L. cincinnatus* sequences exhibited high levels of molecular variation (*k* = 6.5) (Appendix, Fig. 1B) reflecting the already documented intragenomic ITS divergence in this species (Lindner and Banik 2011). Another *Laetiporus* species (*L. huroniensis*) that was poorly sampled by Lindner and Banik (2011) showed similarly high levels of molecular variation (*k* = 7.3). These two species displayed three nonsingleton 97% operational taxonomic units (OTU) (Fig. 1D) and numerous subgroups in the ITS phylogenies (Fig. 2). With a few exceptions, the remaining species displayed low sequence variation primarily with *k* < 1 and one nonsingleton 97% OTU. However, in addition to the two *Laetiporus* species, four additional species (*Armillaria* cf. *novae-zelandiae*, *Aspergillus* sp., *Annulohypoxylon multiforme*, and *Polyporales sp*.; see Fig. 1D) displayed more than one nonsingleton OTU at 97% sequence identity (Appendix).

Four species (*Aspergillus* sp., *Annulohypoxylon multiforme*, *L. cincinnatus*, and *L. huroniensis*) displayed signs of intragenomic ITS variation when neighbor-joining trees were constructed; the remaining species displayed star-shaped trees (unrooted) more suggestive of PCR and pyrosequencing error (Fig. 2). One species, *Saccharomyces cerevisiae*, displayed a very high number of divergent sequences at both the 99% and 97% levels, although the vast majority of these sequences were singletons (Fig. 1C and E) and the unrooted neighbor-joining tree for this species was star shaped (Fig. 2).

## Discussion

Although three of the 98 previously unsampled fungal species (≈3%) displayed signs of intragenomic variation in the ITS region based on neighbor-joining analyses and five species (≈5%) displayed greater than one nonsingleton cluster at 97% sequence identity, the majority of species displayed levels of sequence variation that likely could be ascribed to PCR and sequencing errors. Hence, most species seem to possess well-homogenized ITS tandem arrays, indicating that intragenomic variation in the ITS region will not severely affect environmental studies utilizing next-generation sequencing if certain data-handling steps are followed. In our dataset of 98 taxa (excluding the positive control *L. cincinnatus*), 97% clustering of sequences produced 281 OTUs (187% inflation), whereas similar clustering with the exclusion of singletons produced 107 OTUs (9% inflation) (Appendix). Our results support removal of all singleton clusters in addition to sequence denoizing (Quince et al. 2011) as critical steps for limiting taxon inflation due to PCR/sequencing errors and/or intragenomic variation.

Interestingly, one species, *Saccharomyces cerevisiae*, showed an abundance of divergent singleton sequences (42 of 43 OTUs were singletons at 97% clustering), although without greater sequencing depth it is difficult to say if these divergent singletons are due to PCR/sequencing errors or intragenomic variability. Unfortunately it is difficult to distinguish among PCR/sequencing errors and intragenomic variation, although in the case of PCR/sequencing errors the number of haplotypes should increase with sequencing depth, whereas for intragenomic variability there should be little correlation between the number of haplotypes and sequencing depth (cf. Dickie 2010). With great enough sequencing depth, it should be possible within individual species to distinguish between plateauing/stabilizing numbers of intragenomic haplotypes and sequencing errors.

Additional methods specifically correcting for inflation due to intragenomic variability could be developed as next-generation sequencing methods are employed to screen larger numbers of taxa for the presence of intragenomic heterogeneity and rare ITS paralogs are documented. For traditional Sanger-based sequencing projects utilizing consensus sequences (e.g., from root tips, fruiting bodies, or cultures), the presence of rare ITS haplotypes in a genome does not appear to be a major concern, given that species with significant intragenomic ITS variation (e.g., *L. cincinnatus*) can produce “clean” consensus sequences representing the most common ITS variants (Lindner and Banik 2011). However, if certain variants in an ITS array become common, these copies could manifest themselves as seemingly unresolvable bases in sequence chromatograms, a phenomenon observed when allelic heterozygosity in ITS is encountered as a result of differing nuclei in a dikaryotic/heterokaryotic mycelium (Huang et al. 2010; Hyde et al. 2013). In the case of allelic heterozygosity of ITS, one would expect to observe two primary variants in approximately equal ratios.

For the fungal strains used in this study, we cannot entirely rule out that some of the observed variation is due to differing nuclei within a single mycelium (see Horton 2006), despite the fact that efforts were made to ensure monokaryotic isolates (e.g., sampling cultures derived from single spores and screening all Basidiomycota for the presence of clamp connections, a morphological feature indicative of a dikaryon). For species containing intragenomic variation (e.g., *Annulohypoxylon multiforme*, *Aspergillus* sp., *L. cincinnatus*, and *L. huroniensis*; Fig. 2), we observed more than two variants/clades and variants were not observed in approximately equal ratios, as would be expected if this variation was due to heterozygosity in a dikaryotic/heterokaryotic mycelium. In addition, the level of variation observed for some species was very high, with k (average nucleotide difference) ranging up to 7.4 in *L. huroniensis*. Such a high level of allelic divergence is typically not expected in a heterozygous individual (Hughes et al. 2009).

In order to fully understand the extent of intragenomic ITS variation in fungi, a broader phylogenetic range of species will need to be surveyed, including members of other fungal phyla such as Chytridiomycota s.l., Glomeromycota, and Zygomycota s.l. Significant intragenomic ITS variation has recently been detected in *Batrachochytrium dendrobatidis*, the chytrid fungus implicated in worldwide amphibian declines (Berger et al. 1998). Individual *B. dendrobatidis* genomes were found to contain up to 20 ITS haplotypes per genome (Schloegel et al. 2012), suggesting that significant intragenomic variation in the ITS region is a phenomenon that occurs in diverse fungal lineages. Our results indicate that high-throughput sequencing works well for detecting intragenomic variation and could be applied to an even wider range of species, although it will be difficult to screen fungi that are difficult to culture (e.g., Glomeromycota) or for which haploid material may be difficult to obtain. Given an estimated 1.5–5.1 million fungal species worldwide (Hawksworth 2001; Schmit and Mueller 2007; Blackwell 2011), few generalizations can be made because at best approximately 0.01% of fungal species have been sampled for intragenomic variation to date.

Because fungi are extremely diverse and it is difficult and time consuming to characterize species using traditional methods, it has recently been suggested that fungal species could be formally named based on environmental ITS data (Hibbett et al. 2011). Given the current findings, formal naming of environmental ITS sequences may present potential problems that need to be taken into account because environmental sequences will not always correspond to species in the traditional sense. Reconciling disparate ITS copies under one and the same species would be possible under such systems, but would require prior knowledge and manual intervention. Despite these potential problems, the ITS region seems to be the best DNA barcode currently available, although additional regions will be needed in the future for many fungal groups (cf. Gazis et al. 2011).

The present work suggests that significant intragenomic variation in the ITS region is potentially widespread in a small percentage of species throughout kingdom Fungi. A possible mechanism for generation of intragenomic variation is hybridization (James et al. 2009), although mechanisms capable of maintaining this variation are poorly understood. Understanding the mechanisms that allow ITS paralogs to “escape” concerted evolution in certain species may be the key to understanding how concerted evolution acts so efficiently in the majority of situations. Despite the fact that a large percentage of eukaryotic DNA is repetitive and subject to homogenization via concerted evolution, the fundamental mechanisms of concerted evolution remain largely unknown (Dover 1993; Elder and Turner 1995; Liao 1999).

Given that ITS regions often differ among species, it must be concluded that the ITS region typically evolves significantly during the time it takes for species to diverge. However, it is not known if species displaying large levels of intragenomic variation are being “caught in the act” of evolving, or whether these species can maintain this variation over long evolutionary periods of time. When ITS sequences diverge via speciation, the observed variation will be based on a combination of how quickly species diverge (i.e., how quickly the ITS regions diverge) relative to how quickly concerted evolution erases variation. If rare ITS paralogs do indeed represent traces of previous speciation or hybridization events, it may be possible to use these variants to better understand the evolution of fungal species complexes.

## Appendix

**Table:** List of single-spore isolates subjected to pyrosequencing and summary of molecular variation statistics by isolate

Taxon	Isolate code/Collection number	Number of sequences after initial data filtering	Number of sites	Number of polymorphisms	Number of haplotypes	Haplotype diversity	Nucleotide diversity (Pi)	Average nucleotide difference (k)	Number of clusters (OTUs) at 97% sequence identity	% of total # of sequences in the most abundant 97% cluster	Number of nonsingleton clusters (OTUs) at 97% sequence identity	Number of clusters (OTUs) at 99% sequence identity	Number of nonsingleton clusters (OTUs) at 99% sequence identity
*Aleurodiscus oakesii*	HHB-14363	596	338	58	52	0.218	0.00164	0.444	1	100.00	1	11	1
*Alternaria alternata*	VI 04067	343	294	33	32	0.194	0.00132	0.337	1	100.00	1	6	1
*Annulohypoxylon multiforme*	1967-10 ss-1	903	349	70	71	0.44	0.01582	3.607	3	99.67	2	26	4
*Antrodia carbonica*	MJL-1364	1775	398	98	97	0.159	0.00107	0.243	2	99.94	1	32	1
*Armillaria* cf. *novae-zelandiae*	HHB-15567	2588	563	117	148	0.1557	0.00083	0.216	16	98.96	3	107	4
*Ascocoryne cylichnium*	VI 03548	302	365	65	41	0.282	0.00339	1.004	2	99.67	1	14	1
*Aspergillus* sp.	VI 05307	383	323	54	46	0.492	0.01927	4.97	5	86.68	4	16	8
*Asterostroma cervicolor*	French	1274	357	81	95	0.558	0.00335	0.777	3	99.84	1	39	1
*Auriporia aurulenta*	HHB-8864	2753	410	99	128	0.1581	0.00133	0.277	1	100.00	1	43	1
*Bipolaris sorokiniana*	VI 05062	319	319	68	35	0.283	0.00324	0.821	2	99.69	1	8	1
*Bjerkandera adusta*	FP-101729	380	339	58	52	0.349	0.00247	0.703	1	100.00	1	18	1
*Botryotinia calthae*	1907.3	239	262	21	18	0.168	0.00106	0.258	1	100.00	1	2	1
*Candida albicans*	VI 05304	2156	283	78	88	0.129	0.00105	0.223	1	100.00	1	11	1
*Cenangium ferruginosum*	2678.P	2764	350	107	135	0.1588	0.00116	0.258	2	99.96	1	24	2
*Ceraceomyces americanus*	HHB-11668	176	325	35	26	0.48	0.00477	1.431	1	100.00	1	3	1
*Ceratocystis polonica*	1997-770/9	1594	321	84	76	0.146	0.00126	0.274	2	99.94	1	23	1
*Ceriporiopsis subvermispora*	L-14807	1274	358	84	88	0.217	0.00159	0.409	1	100.00	1	20	1
*Cerocorticium rickii*	FP-102045	284	399	72	50	0.361	0.00343	1.027	2	99.65	1	21	1
*Chlorociboria aeruginosa*	1785.P	3205	338	100	141	0.2178	0.00194	0.378	1	100.00	1	28	2
*Chrysosporium guarri*	VI 05044	1843	379	81	86	0.125	0.00078	0.205	2	99.95	1	16	1
*Ciboria acerina*	1955.1	2821	318	87	118	0.1419	0.00104	0.208	4	99.89	1	32	1
*Cladosporium sp*	Marie6	1587	311	77	76	0.135	0.00092	0.209	2	99.94	1	12	1
*Claviceps purpurea*	VI 04914	1798	355	85	94	0.174	0.00158	0.321	4	99.83	1	34	2
*Collybia carleae*	GB-263.02	949	364	64	77	0.242	0.00148	0.382	1	100.00	1	24	1
*Cotylidia undulata*	HHB-13581	1241	422	82	100	0.22	0.00142	0.374	2	99.92	1	27	1
*Cryptoporus volvatus*	CORAM-74-13	1049	354	95	104	0.2752	0.00185	0.489	1	100.00	1	19	1
*Cudonia confusa*	2487.P	975	275	60	58	0.152	0.00138	0.297	1	100.00	1	8	1
*Cylindrobasidium albulum*	HHB-15055	515	415	78	75	0.411	0.00281	0.804	2	99.81	1	44	1
*Cyptotrama aspersa*	DR-58	1341	506	95	114	0.2931	0.0022	0.588	5	99.70	1	98	1
*Debaryomyces hansenii*	VI 05047	1357	356	82	79	0.161	0.00118	0.306	1	100.00	1	14	1
*Dermea balsamea*	1980-50	475	293	42	30	0.146	0.00107	0.281	1	100.00	1	6	1
*Diaporthe eres*	2003-182	1297	342	105	105	0.219	0.00219	0.472	4	99.77	1	45	1
*Dichostereum effuscatum*	FP-101758	227	331	58	30	0.255	0.00259	0.755	1	100.00	1	7	1
*Echinodontium tinctorium*	Aho-80	1330	340	104	95	0.199	0.00141	0.371	2	99.92	1	20	1
*Entoleuca mammata*	3635.P	1187	263	70	56	0.143	0.00116	0.245	2	99.92	1	8	2
*Fomitopsis pinicola*	32-TT	1215	391	101	122	0.3028	0.00245	0.616	3	99.84	1	39	1
*Fusarium graminearum*	VI 05256	1595	295	62	66	0.138	0.00095	0.208	2	99.94	1	13	1
*Fusarium* sp.	Marie4	1136	282	64	62	0.128	0.00102	0.243	1	100.00	1	7	1
*Geopyxis carbonaria*	1805.1	2221	357	103	104	0.1928	0.00149	0.368	1	100.00	1	18	1
*Gloeophyllum mexicanum*	FP-104037	536	316	63	52	0.215	0.00147	0.38	2	99.81	1	14	1
*Hapalopilus croceus*	HHB-10800	1785	522	106	107	0.223	0.0016	0.387	6	99.72	1	110	1
*Hericium abietis*	Aho-42	1553	354	82	89	0.179	0.00124	0.286	4	99.81	1	35	1
*Hohenbuehelia mastrucata*	TJV-92-1	1280	438	116	118	0.2892	0.00223	0.623	3	99.84	1	35	1
*Hymenoscyphus rhodoleucus*	2329.P	2180	330	97	103	0.1174	0.00085	0.184	1	100.00	1	23	1
*Hyphodontia arguta*	FP-101727	2147	407	84	97	0.153	0.00105	0.228	3	99.91	1	50	1
*Hypochnicium vellereum*	HHB-10134	1071	417	113	98	0.295	0.0021	0.609	9	99.25	1	35	1
*Inonotus circinatus*	FP-102449	604	363	97	74	0.471	0.00316	0.92	2	99.83	1	11	1
*Junghuhnia nitida*	FP-104355	1194	359	102	103	0.247	0.00166	0.431	1	100.00	1	24	1
*Lachnum virgineum*	2802.P	795	313	68	53	0.171	0.0015	0.376	3	99.75	1	16	1
*Laeticorticium roseum*	Weholt-72	910	329	67	72	0.217	0.00161	0.383	1	100.00	1	27	1
*Laetiporus cincinnatus*	HHB-15746	2387	375	99	193	0.6829	0.03681	6.515	5	82.49	3	91	25
*Laetiporus conifericola*	AK-1	2041	334	106	126	0.1898	0.00148	0.349	1	100.00	1	23	2
*Laetiporus huroniensis*	HMC-1	4159	376	122	216	0.56	0.0402	7.356	6	72.47	3	52	9
*Laetiporus sulphureus* s.l.	DA-41	1321	312	85	73	0.493	0.00272	0.65	2	99.92	1	11	1
*Laxitextum bicolor*	NO-7316	2515	346	96	105	0.135	0.00081	0.18	2	99.96	1	26	1
*Lentinus tigrinus*	466	1385	363	79	92	0.194	0.00134	0.349	1	100.00	1	19	2
*Leptodontidium beauverioides*	1995-593/61	711	312	66	57	0.169	0.00149	0.343	1	100.00	1	20	2
*Marasmius fiardii*	PR-910	625	391	83	87	0.339	0.00291	0.693	6	99.20	1	57	1
*Monilinia oxycocci*	1910.3	1176	288	75	78	0.171	0.00122	0.278	2	99.91	1	6	1
*Nemania serpans*	VI 05113	1098	318	83	76	0.192	0.00147	0.374	1	100.00	1	12	1
*Neofabraea krawtzewii*	1966-65/5 ss-2	1793	306	92	100	0.148	0.00099	0.231	1	100.00	1	14	2
*Neolentinus lepideus*	HHB-14362	1633	356	84	94	0.381	0.0022	0.49	1	100.00	1	47	1
*Neonectria ditissima*	1961-67 ss1-1	731	276	63	52	0.182	0.00152	0.338	3	99.73	1	7	1
*Omphalotus olearius*	HHB-17222	1663	435	80	96	0.211	0.0015	0.357	4	99.82	1	55	1
*Panellus serotinus*	HHB-11719	1110	441	101	117	0.3599	0.00258	0.726	4	99.73	1	42	1
*Pestalotiopsis* sp.	Marie7	778	366	73	59	0.211	0.0016	0.428	2	99.87	1	13	2
*Fuscoporia gilva*	HHB-11806	915	401	89	75	0.195	0.00132	0.38	2	99.89	1	20	1
*Phlebia acerina*	GB-0568	708	362	70	72	0.265	0.00181	0.486	2	99.86	1	12	1
*Pleurotus ostreatus*	FP-70837	1322	424	123	106	0.2339	0.0015	0.398	1	100.00	1	41	1
*Plicaria endocarpoides*	1802.1	413	337	68	47	0.337	0.00325	0.918	2	99.76	1	10	1
Polyporales sp.	HHB-9461	2021	386	90	116	0.1871	0.0015	0.324	8	99.55	2	52	1
*Polyporus brumalis*	FP-102443	1221	402	128	92	0.191	0.00182	0.487	3	99.84	1	22	1
*Pseudallescheria boydii*	VI 03873	1605	335	85	105	0.1882	0.00157	0.324	2	99.94	1	38	2
*Pycnopeziza sympodialis*	2404.P	3546	369	112	149	0.136	0.00119	0.239	3	99.94	1	49	1
*Rhodotus palmatus*	FP-105911	993	432	118	98	0.269	0.00198	0.597	1	100.00	1	30	1
*Saccharomyces cerevisiae*	VI 05279	698	620	98	163	0.6993	0.00683	2.171	43	93.98	1	444	5
*Sarcoscyphus coccinea*	2346.P	1522	334	109	146	0.7133	0.00624	1.324	1	100.00	1	28	2
*Schizophyllum commune*	NO-7599	271	273	30	22	0.176	0.00123	0.302	1	100.00	1	5	1
*Sclerotinia carpii*	2195.2	2136	308	78	88	0.102	0.0007	0.156	1	100.00	1	10	1
*Sclerotinia tetraspora*	1993.1	2416	315	73	76	0.119	0.0011	0.216	1	100.00	1	22	2
*Scutellinia scutellata*	1968-138/1	2297	411	123	168	0.2768	0.00153	0.383	4	99.87	1	43	1
*Scytinostroma galactinum*	FP-101874	437	339	63	46	0.216	0.00194	0.565	1	100.00	1	7	1
*Serpula himantioides*	FP-97419	1341	418	113	102	0.2045	0.00179	0.409	4	99.78	1	39	1
*Sirococcus strobilinus*	93-298/67/2	1584	359	112	115	0.224	0.00174	0.426	1	100.00	1	16	1
*Sistotrema brinkmanii*	HHB-7604	2119	349	85	83	0.122	0.00109	0.231	3	99.91	1	36	1
*Sistotremastrum suecicum*	HHB-10207	1911	366	92	106	0.1547	0.00106	0.231	2	99.95	1	46	2
*Steccherinum laeticolor*	HHB-13083	1747	369	97	118	0.1912	0.00159	0.355	4	99.83	1	59	2
*Stereum hirsutum*	FP-91666	3051	387	104	119	0.1114	0.00071	0.155	1	100.00	1	44	1
*Strasseria geniculata*	1975-4	2019	330	120	139	0.2901	0.002	0.444	3	99.90	1	30	1
*Trametes pubescens*	L-16013	1436	342	92	95	0.179	0.0015	0.362	1	100.00	1	32	1
*Trechispora polyporoidea*	L-8920	2817	381	95	115	0.133	0.00085	0.189	2	99.96	1	31	1
*Trichaptum biforme*	FP-50251	692	427	110	78	0.261	0.00209	0.64	3	99.71	1	30	1
*Trichoderma hamatum*	VI 05221	2151	354	86	89	0.162	0.00146	0.333	3	99.91	1	19	1
*Tympanis hypopodia*	1978-433/1	3967	341	106	123	0.1201	0.00088	0.176	3	99.95	1	22	1
*Tyromyces chioneus*	FP-103224	1755	372	95	118	0.2824	0.00276	0.614	4	99.83	1	35	1
*Veluticeps berkeleyi*	SD-62-6-4	1831	397	101	107	0.178	0.00128	0.294	2	99.95	1	35	1
*Vibrissea truncorum*	3169.P	2354	328	96	94	0.122	0.00083	0.188	2	99.96	1	16	1
*Wrightoporia cylindrospora*	FP-90117	4212	400	113	171	0.1631	0.00124	0.227	4	99.93	1	89	2
*Zalerion arboricola*	1968-2/12	1890	320	78	88	0.143	0.00085	0.202	1	100.00	1	11	1
	Total:	148046			9086				286		110	3241	163
